# The health-related determinants of eating pattern of high school athletes in Goiás, Brazil

**DOI:** 10.1186/s13690-020-0396-3

**Published:** 2020-03-12

**Authors:** Matias Noll, Ana Paula Santos Rodrigues, Erika Aparecida Silveira

**Affiliations:** 1 Instituto Federal Goiano – Campus Ceres, Ceres, Brazil; 2 Programa de Pós-Graduação em Ciências da Saúde, Faculdade de Medicina, Universidade Federal de Goiás, Goiânia, Brazil

**Keywords:** Adolescent, Sport, Health, Eating behavior, Food

## Abstract

**Background:**

Athletes put their health at short- and long-term risk and a detailed investigation of health outcomes is important because it should allow the development of more specific interventions. This study aimed to evaluate the health-related determinants of eating pattern of high school athletes.

**Methods:**

The study enrolled 248 Brazilian athletes aged 14–20 years. Eating pattern outcomes included skipping breakfast, vegetable and fruit consumption, and sweets consumption. The following factors were considered as independent variables: sociodemographic, economic, anthropometry, body weight control, psychosocial, sedentary behavior, sleeping time, meals, alcohol and smoking, and strength variables. Data were analyzed using Poisson regression model and the effect measure was the prevalence ratio (PR).

**Results:**

The results showed that 45.6% of the athletes skipped breakfast, 29.8% ate sweets regularly, and only 8.9% consumed vegetables and fruit regularly. Multiple analysis revealed the following positive associations: breakfast skipping and vomiting or using laxatives to lose weight (PR, 1.12; 95% CI, 1.01–1.26); low fruit and vegetable consumption and using pills to lose weight (PR, 1.05; 95% CI, 1.02–1.07); high sweets consumption and female athletes (PR, 1.32; 95% CI, 1.12–1.55); high sweets consumption and more than 2 h spent watching TV (PR, 1.19; 95% CI, 1.02–1.39).

**Conclusions:**

Our findings indicated that sociodemographic, body weight control and sedentary factors are determinants on eating patterns of high school athletes.

## Background

Engaging in sports is an important way to reduce a sedentary lifestyle, increase physical activity levels, and promote healthy habits [1]. Active people are at lower risk for many health-related conditions such as cardiovascular disease, diabetes, obesity, cancer, poor skeletal health, poor quality of life, and mental health [2, 3]. Therefore, sports participation is stimulated, both within and outside the school environment. Recent mega events in Brazil such as the 2014 FIFA World Cup, 2015 Pan American Games, and 2016 Olympic and Paralympic Games, may have generated considerable interest in sporting activities and possibly encouraged exercising in the young population. Moreover, programs developed by the Brazilian Ministry of Sports, such as the *Programa Segundo Tempo* (“Second Half Program”), have provided access to sports practice for more than 4 million adolescents [4].

Despite the health benefits of sports participation, it poses risks for young athletes [1] such as alcohol consumption, smoking, vomiting or using laxatives, taking weight-loss pills, and using performance-enhancing substances [5–7]. These behaviors may considerably preclude the chances of sporting success and reduce both career longevity and health in adulthood [8]. Moreover, the eating pattern is one of the factors that have received attention because of its relationship with sports performance. Thus, the same attention given to increase sport participation should be given to the health of young athletes as well to the risks brought about by sports.

Scaglioni et al. [9] described that the eating pattern involves a complex interaction of genetic, familial, and environmental factors. Although the relationship between eating patterns and health has been underestimated, and most guidelines treat foods as mere nutrient carriers [10–12], current investigations have extended beyond the simple intake of nutrients to consider food choice as well as its level of processing [13, 14]. However, recent systematic review with young athletes [15] showed that most studies still focus on nutrient analysis and there is a gap regarding eating patterns studies and its associate factors. Recent study [16] found that young athletes who practice sports for many years have a more regular healthy food consumption. Nevertheless, the relationship between health outcomes and eating patterns was underestimated [12, 15].

It is necessary to monitor the eating patterns of young athletes since their attitudes to meals and food choice can impact their training and performance, as well as their optimal growth and development [17]. Currently, skipping breakfast, fruit and vegetable consumption, and sweets consumption are key determinants of a healthy diet, and health factors related to these outcomes have been investigated [17, 18]. Moreover, the health-related determinants of eating pattern of young athletes have not been well studied in emerging countries [15].

Athletes put their health at short- and long-term risk [5] and a detailed investigation of health outcomes is important because it should allow the development of more specific interventions. Thus, our study aimed to evaluate the health-related determinants of eating pattern in Brazilian high school athletes. We improved the assessment of health-related determinants, i.e. to evaluate common outcomes such as anthropometry, sedentary behavior, alcohol consumption, and smoking; and also included poorly assessed factors such as psychosocial factors, body weight control, and trunk and handgrip strength.

## Methods

The present cross-sectional study was based on data from the ‘Brazilian High School Athlete Study: Health-related Outcomes’, a study performed during the state phase of the 2015 Federal Institutes Games (FIGs) in Brazil [16, 19, 20]. These games are organized annually and the state phase, in which the top-ranked athletes are selected, is followed by the regional and national phases. In 2015, the FIGs occurred in the Urutaí city, state of Goiás, Brazil.

Representing 12 cities of the state of Goiás in the Brazilian Midwest, a total of 361 high school athletes regularly enrolled in the Federal Institute participated in the FIG. Athletes were included based on the following inclusion criteria: age of 14–20 years, no previous history of musculoskeletal surgery, and participation in one of the following sports – handball, basketball, volleyball, or soccer. Pregnancy, injured athletes, as well as those who declined to participated were excluded from the study.

Athletes were invited to participate by contacting coaches 1 week before the tests as well as by posters fixed in the competitive environment. All athletes participating in the FIGs were recruited to the present study, and 320 athletes were included. Twenty-seven athletes were injured and 42 declined to participate, thus, the study enrolled 251 athletes. All tests and questionnaires were performed individually in a properly equipped room near the competition environment.

An overview of the measures, classification parameters, and dichotomization, used in this study, are presented in the Additional file 1: Table S1. The Brazilian National School-Based Health Survey (PeNSE), a reproducible self-administered questionnaire [21], which was validated using 24-h recall, was used to assess the following variables: sociodemographic, economic, body weight control, psychosocial, and alcohol consumption and smoking.

The PeNSE was also used to evaluate the frequency of eating, within the previous 7 days, of vegetable, fruits, and sweets, and how often the athlete had breakfast. These variables were evaluated using the following questions: “In the last 7 days, on how many days did you eat at least one type of raw or cooked vegetable?”, “In the last 7 days, on how many days did you eat fresh fruits or fruits salad?”, “In the last 7 days, on how many days did you eat sweets?”, and “In the last 7 days, on how many days did you have breakfast?”. The eight available response options were “I did not eat in the last 7 days,” “I ate on 1 day (2, ..., 6 days) of the last 7 days,” and “I ate on all of the last 7 days” [21]. For data analysis purposes, they were categorized as regularly consumed (for at least 5 days in the previous 7 days) or not [22–26]. All athletes are regularly enrolled in school and study in the mornings, so we consider breakfast between 6 and 8:59 am [27]. At the weekend it was considered the same time for breakfast.

At the outset of the study, the researcher explained to the subjects in a group meeting how the questionnaire should be answered. The subjects then answered the questionnaires individually. The PeNSE reliability was tested on 34 high school students by the 7-day test-retest protocol. All the questions indicated good and very good values (Kappa range, 0.701–0.841) [16].

The short version of the International Physical Activity Questionnaire (IPAQ) validated for the Brazilian population was used to evaluate sedentary behavior [28]. Sedentary behavior was assessed using the short-IPAQ sitting item that consisted of a single question regarding the time spent on activities such as studying, reading, watching television (TV), computer use, and other sitting or reclining activities in a weekday and at weekends. Excessive sitting time was classified as sitting time ≥ 9 h daily. This threshold was consistent with the highest quintile of IPAQ-assessed sitting time in the 20-country comparison of the descriptive epidemiology of sitting. Moreover, to determine the time spent sleeping, watching television, and using computers, a self-administered questionnaire entitled “Back Pain and Body Posture Evaluation Instrument” (BackPEI) was used [29].

Each athlete’s body mass and height were measured. Students were wearing light clothes at the time of data collection and were instructed to remain standing, upright, with the face directed forward, and shoulders relaxed. For height and body weight measurement, we used a stadiometer (accuracy, 0.1 mm) and a digital scale (Plenna-MEA-03140, São Paulo, Brazil; maximum capacity, 150 kg; accuracy, 100 g), respectively. Body mass index (BMI) in kg/m^2^ was calculated by dividing the mass (kg) by the squared height (m^2^).

Handgrip strength was measured with the athlete sitting, elbow flexed at 90°, shoulders adducted and neutrally rotated, forearm in the neutral position, and the wrist 10° extended [30]. Trunk strength was measured with the athlete standing, legs kept straight and the back flexed 45°. Each athlete performed two isometric valid trials (5 s) for the trunk and right and left-hand strength, with a 60-s rest period between trials, and the largest value was registered. The average of both handgrip strengths was considered. Trunk and handgrip strength were normalized by body weight.

For both strength tests, participants were encouraged to apply their maximum strength. Handgrip strength was evaluated using a hand dynamometer (EMG SYSTEM, TRF_MAN200 model, São José dos Campos, Brazil), and the trunk strength by a lumbar dynamometer (EMG System, TRF_ELMB200 model, São José dos Campos, Brazil). The dynamometers (nominal capacity 200 kg, sensitivity 2 mV/V ± 10%, error < 0.03%, input resistance 405 Ω, output resistance 350 Ω) were calibrated before data collection and were adjusted according to the size of each athlete.

The test-retest reliability of the questionnaires and strength tests were good as shown in our previous study [20]. The present study was in accordance with the Helsinki Declaration and was approved by the Ethics Committee for Human Research of the Instituto Federal Goiano. Athletes and their parents or guardians, in the case of minors, voluntarily signed the informed consent form.

Data were analyzed using descriptive statistics and the Wald Chi-Square test of association (bivariate analysis) for the eating pattern outcomes: skip breakfast, irregular vegetable and fruit consumption, and regular sweet consumption. The following factors were considered independent group variables: sociodemographic, economic, anthropometry, body weight control, psychosocial, sedentary behavior, sleeping time, meals, alcohol and smoking, and strength variables. These variables are shown in the Additional file 1: Table S1.

Independent variables with a level of significance of *p* < 0.20 in the bivariate analysis were included in a multiple analysis using the Poisson regression model with robust variance. The effect measure was the prevalence ratio (PR) with its respective 95% confidence intervals (CIs), and the reference category was selected to verify negative factors. We adjusted these analyses for sociodemographic (gender and age) variables (α = 0.05). Statistical analyses were performed using the Statistical Package for the Social Sciences (SPSS 20.0, IBM, Armonk, NY, USA).

## Results

Of the 251 athletes enrolled, 248 (98.8%) completed the protocol. Of these, 144 (58.1%) were aged 14–16 years and 104 (41.9%) aged 17–20 years, and most were boys (68.5%).

**Fig. 1:**
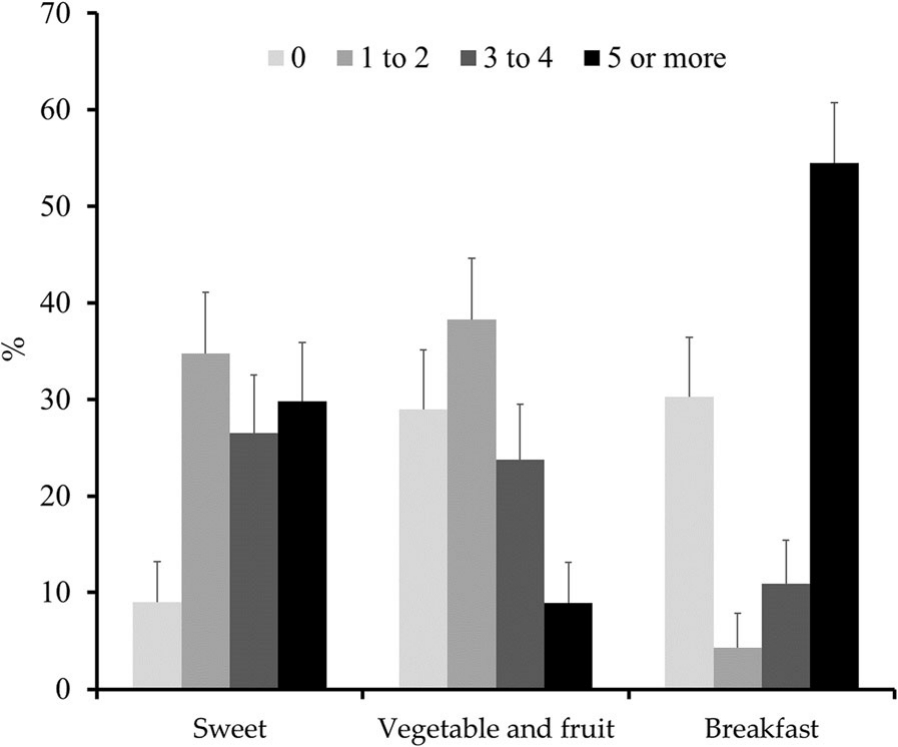
Prevalence of eating pattern outcomes per week

The prevalence of outcomes is presented in Fig. 1. Of all the athletes, 54.5, 29.8, and 8.9% had breakfast, ate sweets, and consumed vegetables and fruit regularly, respectively.

The high prevalence of athletes that felt lonely and lost sleep because of a concern, engaged in sedentary behavior, whose time sleeping per night was less than recommended, and who were involved with alcohol consumption and passive smoking, may be noted in Table 1.

**Table 1:** Prevalence of health-related determinants

Variables	Total N (%)	Skip breakfast ^a^ n (%)	Low vegetable and fruit consumption ^a^ n (%)	High sweets consumption ^b^ n (%)
Sociodemographic				
Age				
14–16 years	144(58.1)	62(43.1)	136(94.4)	43(30.3)
17–20 years	104(41.9)	51(49)	90(85.6)	30(29.1)
Sex				
Male	170(68.5)	79(46.5)	158(92.9)	34(20.2)
Female	78(31.5)	34(43.6)	68(87.2)	39(50.6)
Economic				
Mother education level				
Basic education	53(23.1)	23(43.4)	47(88.7)	16(30.2)
High school/College	176(76.9)	82(46.6)	163(92.6)	49(28.3)
Have a part time job				
No	219(88.7)	98(44.7)	199(90.9)	67(31)
Yes	28(11.3)	14(50)	26(92.9)	6(21.4)
Anthropometric				
Body mass index				
Normal	180(73.5)	82(45.6)	162(90)	55(30.6)
Overweight	65(26.5)	30(46.2)	61(93.8)	18(29)
Body weight control				
Vomiting or using laxatives to lose weight				
No	220(95.2)	99(45)	199(90.5)	65(30)
Yes	11(4.8)	7(63.6)	10(90.9)	6(54.4)
Using pills to lose weight				
No	227(96.6)	105(46.3)	205(90.3)	70(31.2)
Yes	8(3.4)	3(37.5)	8(100)	2(25)
Using pills to improve muscle mass				
No	217(92.3)	100(46.1)	196(90.3)	69(32.2)
Yes	18(7.7)	8(44.4)	17(94.4)	3(16.7)
Psychosocial				
Body weight self-perception				
Normal	130(55.6)	61(46.9)	113(86.9)	40(31)
Not normal	104(44.4)	47(45.2)	99(95.2)	32(31.4)
Felt lonely last year				
Never and Rarely	162(68.9)	72(44.4)	146(90.1)	50(31.4)
Sometimes or more	73(31.1)	36(49.3)	67(91.8)	22(30.1)
Lost sleep because of a concern last year				
Never and Rarely	171(73.1)	79(46.2)	154(90.1)	44(26.2)
Sometimes or more	63(26.9)	28(44.4)	58(92.1)	27(42.9)
Felt intimidated last month				
Never and Rarely	199(82.6)	89(44.7)	191(91)	59(30.1)
Sometimes or more	42(17.4)	22(52.4)	38(90.5)	14(33.3)
Sedentary Behavior				
Time spent watching TV				
0–1 h per day	143(62.2)	65(45.5)	127(88.8)	32(22.5)
≥ 2 h per day	87(37.8)	41(47.1)	82(94.3)	32(37.6)
Time spent using computer				
0–1 h per day	105(49.8)	40(38.1)	98(93.3)	27(26.2)
≥ 2 h per day	106(50.2)	53(50)	96(90.6)	32(30.2)
Sitting time on a weekday				
0–8 h per day	142(59.9)	66(46.5)	131(92.3)	38(27.3)
≥ 9 h per day	95(40.1)	42(44.2)	86(90.5)	34(35.8)
Sitting time on a weekend day				
0–8 h per day	99(42.1)	72(43.6)	150(90.9)	52(32.1)
≥ 9 h per day	136(57.9)	34(48.6)	65(92.9)	19(27.1)
Meals				
Have regular breakfast				
No	113(45.6)	–	104(92)	37(33.3)
Yes	135(54.4)	–	122(90.4)	36(26.9)
Habit of eating in front of the TV				
No	98(39.5)	44(44.9)	86(87.8)	21(21.6)
Yes	150(60.5)	69(46)	140(93.3)	52(35.1)
Sleeping time				
Time sleeping per night				
≤ 7 h	163(72.4)	79(48.5)	151(92.6)	47(29.4)
≥ 8 h (Recommended)	62(27.6)	22(35.5)	56(90.3)	21(33.9)
Alcohol and smoking				
Alcohol consumption last month				
No	169(68.1)	73(43.2)	152(89.9)	46(27.7)
Yes	79(31.9)	40(50.6)	74(93.7)	27(34.2)
Frequency of alcohol consumption ^c^				
1 to 5 days last month	51(64.6)	27(52.9)	48(94.1)	16(31.4)
≥ 6 days last month	28(35.4)	13(46.4)	26(92.9)	11(39.3)
Doses of alcohol consumption on any occasion				
1 to 3 doses a time	47(60.3)	24(51.1)	45(95.7)	15(31.9)
≥ 4 doses a time	31(39.7)	15(48.4)	28(90.3)	12(38.7)
Smoking habits last month				
No	233(94)	104(44.6)	213(91.4)	70(30.4)
Yes	15(6)	9(60)	13(86.7)	3(20)
Passive smoking last week				
No	102(42.5)	45(44.1)	94(92.2)	25(25)
Yes	138(57.5)	67(48.6)	124(89.9)	47(34.3)
Smoking parents				
No	189(79.4)	87(46)	173(91.5)	54(29)
Yes	49(20.6)	24(49)	43(87.8)	17(34.7)
Strength				
Trunk strength ^d^				
Low (1.27 ± 0.27)	123(50.2)	54(43.9)	109(88.6)	34(27.9)
High (1.77 ± 0.35)	122(49.8)	58(47.5)	114(93.4)	38(31.7)
Handgrip strength ^d^				
Low (0.45 ± 0.08)	123(50.2)	55(44.7)	109(88.6)	45(36.9)
High (0.71 ± 0.85)	122(49.8)	57(46.7)	114(93.4)	27(22.5)

^a^ Irregular consumption (< 5 days in the previous 7 days); ^b^ Regular consumption (at least 5 days in the previous 7 days)

^c^ Only students that consumed alcohol last month; ^d^ Normalized by body weight

Bivariate analysis examining the relationship between eating habits and health-related determinants are presented in the Table 2. These variables and others with *p* < 0.20 were included in the multiple model.

**Table 2:** Association of eating pattern with sociodemographic, economic, anthropometric, body weight control, psychosocial factors, sedentary time, meals, sleeping time, alcohol and smoke, and handgrip strength variables

	Skip breakfast ^a^		Low vegetable and fruit consumption ^a^		High sweets consumption ^b^	
	PR (95% CI)	*p*	PR (95% CI)	*p*	PR (95% CI)	*p*
Sociodemographic						
Age – 14-16 years	0.98(0.93–1.03)	0.350	**1.03(1.01–1.05)**	**0.042**	1.01(0.88–1.17)	0.845
Sex – female	0.98(0.94–1.04)	0.672	0.98(0.95–1.01)	0.179	**1.43(1.24–1.65)**	**0.001**
Economic						
Mother education level – basic education	0.99(0.93–1.05)	0.682	0.99(0.95–1.02)	0.412	1.02(0.86–1.22)	0.794
Have a part time job – yes	1.02(0.94–1.10)	0.598	1.01(0.97–1.04)	0.704	0.88(0.70–1.10)	0.275
Anthropometric						
Body mass index – overweight	1.01 (0.95–1.06)	0.934	1.01(0.99–1.04)	0.301	0.98(0.83–1.16)	0.821
Body weight control						
Vomit or use of laxatives to lose weight – yes	1.07(0.96–1.20)	0.196	1.01(0.94–1.06)	0.959	1.31(0.98–1.75)	0.071
Pills used to lose weight – yes	0.96(0.84–1.11)	0.621	**1.03(1.02–1.04)**	**0.001**	0.92(0.61–1.39)	0.700
Pills used to improve muscle mass – yes	0.99(0.90–1.09)	0.893	1.01(0.98–1.05)	0.472	0.81(0.62–1.06)	0.126
Psychosocial						
Body weight self-perception – not normal	0.99(0.94–1.05)	0.792	**1.03(1.01–1.05)**	**0.023**	1.01(0.87–1.17)	0.953
Felt lonely last year – sometimes or more	1.02(0.96–1.08)	0.487	1.01(0.98–1.03)	0.677	0.98(0.84–1.15)	0.841
Lost sleep last year – sometimes or more	0.99(0.94–1.05)	0.811	1.01(0.98–1.04)	0.625	**1.22(1.04–1.43)**	**0.014**
Felt intimidated last month – sometimes or more	1.03(0.97–1.10)	0.361	1.00(0.96–1.03)	0.923	1.04(0.86–1.26)	0.682
Sedentary time						
Time spent watching TV per day ≥2 h	1.01(0.95–1.06)	0.805	1.02(0.99–1.04)	0.134	**1.21(1.04–1.40)**	**0.014**
Time spent using computer per day ≥2 h	1.05(0.99–1.11)	0.079	0.99(0.97–1.02)	0.460	1.05(0.90–1.23)	0.523
Sitting time at a weekday ≥9 h	0.99(0.94–1.04)	0.731	0.99(0.97–1.02)	0.645	1.11(0.95–1.29)	0.169
Sitting time at a weekend day ≥9 h	1.02(0.96–1.08)	0.486	1.01(0.98–1.03)	0.608	0.94(0.80–1.10)	0.448
Meals						
Have regular breakfast – no	–		1.01(0.98–1.03)	0.643	1.08(0.94–1.25)	0.271
Habit of eating in front of the TV – yes	1.01(0.93–1.09)	0.865	1.02(0.99–1.05)	0.153	**1.18(1.03–1.37)**	**0.020**
Sleeping time						
Time sleeping per night ≤7 h	1.04(0.98–1.11)	0.187	1.00(0.97–1.03)	0.965	0.99(0.83–1.17)	0.873
Alcohol and smoke						
Alcohol consumption last month – yes	1.03(0.98–1.09)	0.271	1.01(0.99–1.04)	0.298	1.08(0.93–1.26)	0.303
Frequency of alcohol consumption last month – 6 or more	0.97(0.89–1.07)	0.580	0.99(0.96–1.04)	0.830	1.10(0.85–1.42)	0.477
Doses of alcohol on any occasion last month – 4 or more	0.99(0.90–1.08)	0.817	0.98(0.94–1.02)	0.374	1.08(0.84–1.39)	0.536
Smoking habits last month – yes	1.06(0.96–1.17)	0.227	0.98(0.92–1.05)	0.599	0.87(0.65–1.17)	0.362
Passive smoking last week – yes	1.02(0.97–1.07)	0.496	0.99(0.97–1.02)	0.534	1.12(0.97–1.30)	0.120
Parents smoking habits – yes	1.01(0.95–1.08)	0.712	0.99(0.95–1.02)	0.461	1.07(0.90–1.28)	0.445
Strength						
Trunk strength – low	0.98(0.90–1.06)	0.567	0.98(0.96–1.01)	0.201	0.95(0.82–1.10)	0.518
Handgrip strength – low	0.99(0.94–1.04)	0.753	0.98(0.96–1.01)	0.185	**1.20(1.04–1.38)**	**0.013**

Bold *p*-values reflect statistical significance (*p* < 0.05). *PR* Prevalence ratio; 95% *CI* 95% Confidence interval; ^a^ Irregular consumption (< 5 days in the previous 7 days); ^b^ Regular consumption (at least 5 days in the previous 7 days)

After inserting the variables in the multiple analysis, our major findings indicated the following associations: (a) skipping breakfast was associated with vomiting or using laxatives to lose weight; (b) irregular fruit and vegetable consumption with using pills to lose weight; (c) regular sweets consumption with female athletes and with hours spent watching TV (Table 3).

**Table 3:** Adjusted prevalence ratio for eating pattern and sociodemographic, body weight control, psychosocial, sedentary time, meals, sleeping time, alcohol and smoke, and strength variables

	Skip breakfast ^a^		Low vegetable and fruit consumption ^a^		High sweets consumption ^b^	
	Adjusted PR (95% CI)	*p*	Adjusted PR (95% CI)	*p*	Adjusted PR (95% CI)	*p*
Sociodemographic						
Age – 14-16 years			1.03(1.00–1.05)	0.059		
Sex – female			0.98(0.95–1.02)	0.359	**1.32(1.12–1.55)**	**0.010**
Body weight control						
Vomit or use of laxatives to lose weight – yes	**1.12(1.01–1.26)**	**0.036**			1.10(0.88–1.39)	0.390
Pills used to lose weight – yes			**1.05(1.02–1.07)**	**0.001**		
Pills used to improve muscle mass – yes					0.92(0.67–1.26)	0.590
Psychosocial						
Body weight self-perception – not normal			1.02(0.99–1.05)	0.067		
Loss of sleep last year – sometimes or more					1.11(0.94–1.32)	0.195
Sedentary time						
Time spent watching per day ≥2 h			1.02(0.99–1.05)	0.172	**1.19 (1.02–1.39)**	**0.030**
Time spent using computer per day ≥2 h	1.05(0.99–1.11)	0.106				
Sitting time at a weekday ≥9 h					1.04(0.88–1.22)	0.623
Meals						
Habit of eating in front of the TV – yes			1.01(0.98–1.05)	0.343	1.13(0.96–1.33)	0.125
Sleeping time						
Time sleeping per night ≤7 h	1.05(0.99–1.13)	0.099				
Alcohol and smoke						
Passive smoking last week – yes					1.09(0.94–1.27)	0.256
Strength						
Handgrip strength – low			0.99(0.96–1.02)	0.469	1.00(0.84–1.19)	0.760

Multiple analysis according to the Poisson regression model with robust variance. Prevalence ratio (PR) was the effect measure with its respective 95% confidence intervals (CIs). The model was adjusted for sex and age including independent variables (with *p* < 0.20 in the bivariate analysis). Bold *p*-values denote statistical significance (*p* < 0.05); ^a^ Irregular consumption (< 5 days in the previous 7 days); ^b^ Regular consumption (at least 5 days in the previous 7 days)

## Discussion

Our main results indicated the presence of unhealthy behaviors and inappropriate eating patterns in young Brazilian athletes. Moreover, health-related variables are significantly associated with eating pattern: skipping breakfast with vomiting or using laxatives to lose weight; lower fruit and vegetable consumption with using pills to lose weight; and high sweets consumption with two or more hours per day spent watching TV. In emerging countries, research about the effect of health-related determinants on eating pattern is still incipient, and this is the first study with Latin-American high school athletes in this respect [15], providing essential information for more interventions focused on improving unhealthy behaviors.

Our results indicated that approximately half of those assessed skipped breakfast. This prevalence is higher compared with that found in Brazilian adolescents (35%) [31] and around the world (4 to 42%) [32–38]. Recent studies have demonstrated that breakfast is an important meal and is related to several benefits, such as adequate nutrition intake, diet quality and improved exercise performance [35], general health (lower BMI, waist circumference, body fat percentage, and glucose levels) [39], and cognitive and academic performance [40]. Possible reasons for breakfast omission in adolescents athletes include lack of time, poor availability of foods and beverages, and the intention to create an energy deficit to help weight control [40–42]. Although dietary habits that focus on skipping breakfast lead to energy deficit, they may result in unhealthy habits like nibbling snack foods, mainly those high in sugar or fat, culminating in the inability to weight self-control [41].

Restricting nutritional intake in early sports career and weight loss behavior are not recommended and may result in eating disorders [43–45]. In agreement with our findings, a large cross-sectional study of adolescents in the United States demonstrated that vomiting or using laxatives to lose weight (approximately 6%) was positively associated with skipping breakfast [32]. However, regular breakfast consumption is essential to improve performance and is related to the consumption of fiber-rich products that may improve glucose and insulin metabolism, appetite regulation (hunger and satiety), energy balance, and weight control [18]. This implies that regular breakfast helps achieve an ideal body shape and may preclude weight loss behavior.

Our results show that only one-tenth of all athletes regularly consumed vegetables and fruit. These values are much lower than indicated in the literature [33, 46–48]. A study with American adolescent participants in sports [49] found a regular consumption of fruits and vegetables in approximately 50% of young athletes. Currently, it is accepted that a diet rich in fruits and vegetables is associated with lower risks for diabetes, obesity, cardiovascular diseases, and cancer, and was found to be inversely associated with all-cause mortality [50–53]. Although concerning results are expected since Brazilian adolescents have shown unhealthy eating behaviors, with only 30% eating vegetables and fruit regularly [23], our findings indicated a greater problem.

Low vegetable and fruit consumption were associated with weight loss behavior. The present association may be a direct result of using a weight loss medication that suppresses the appetite as well as a non-direct relationship because athletes may want to achieve weight loss by reducing overall energy intake via fruit and vegetable intake restriction. In contrast, results from American youths involved in sports did not show an association between pills intake and fruit and vegetable consumption, but this may be because they were not competitive athletes [54]. Concern is required regarding pills use without prescription to achieve weight loss, because it leads to decreased vegetable and fruit ingestion, and may consequently result in nutrient deficiencies and other adverse health effects, such as eating disorders [17].

Our results show that approximately one-third of all athletes ate sweets regularly. Results from Brazilian athlete [48] and non-athlete youths [22] demonstrated a similar prevalence (25–40%) of regular sweet consumption. Reducing sugar ingestion is essential for health [55], because of the strong association between cardiovascular disease and type 2 diabetes mellitus [56–59]. This is a serious concern because of the characteristics of ultra-processed foods (high energy density, free sugar, sodium, total fat and saturated fat; low protein, and fiber) [60, 61]. Recent studies [13, 62] and the Brazilian Dietary Guidelines [63] advocate for the restriction of foods and beverages with low nutritional value and have recommended a new approach that considers the choice of foods and meals, and the level of processing foods undergo.

Sedentary behavior and the media have a considerable influence on eating patterns [64–67]. Our findings showed that athletes who spent more time watching TV ate more sweets. Moreover, we demonstrated that girls were more likely to consume sweets compared with boys. A greater desire to consume sweets, soft drinks, and fast food may be due to several hours in front of the TV which exposes the viewer to more media advertisements and sedentary behavior that stimulate poor eating habits [25, 66]. These two unhealthy behaviors are a source of greater concern because they are directly related to obesity and other unhealthy behaviors [68].

Although other health-related determinants were not associated with eating patterns, they are also points of concern. Regarding alcohol consumption, the prevalence is high probably because sports are often related with socializing outside the sporting environment and may be greatly influenced by social norms or the traditional relationship between alcohol and sport due to sports sponsorship by beer companies [69]. Decreased training performance due to dehydration and poor recovery may be a consequence of alcohol consumption [38]. On the other hand, smoking had a low prevalence in the present study, possibly because such habit is socially unacceptable [70], especially because in Brazil there was widespread governmental education and preventive programs in the last years. Other health-related behaviors had a high prevalence such as feeling lonely and losing sleep because of a concern, sedentary behavior, less sleeping time, and passive smoking.

Regarding the strengths of the study; first, poorly assessed outcomes such as psychosocial characteristics, body weight control, and handgrip strength were investigated. Second, the investigation of health-related outcomes and their association with eating pattern is essential because health professionals and coaches may be trained to conduct interventions targeted at these unhealthy behaviors. However, some limitations need to be listed: the questionnaire did not evaluate the amount of each food group consumed per day; and, since the food information was self-reported some results could be over or underestimate due to the self-assessment of each athlete and also memory bias. Although there is a consensus in the literature about the importance of implementing intervention programs for young athletes, there is still a lack of knowledge about the eating patterns and their association with health-related behavior. Therefore, our results reinforce the need for education programs improving eating patterns and unhealthy behaviors. These aspects are essential to this critical transition period from adolescence to adulthood.

## Conclusion

High school athletes do not have adequate eating patterns and adequate health-related behavior. Sociodemographic, body weight control and sedentary time are determinant factors of the eating pattern. In addition to evaluating important health-related determinants (i.e. to evaluate common outcomes such as anthropometry, sedentary behavior, substance use); we also have included poorly assessed factors such as psychosocial factors, body weight control, and trunk and handgrip strength. This information is essential to conduct preventive interventions and for improving the sport performance that will benefit athletes throughout their life. Moreover, nutrition and health promotion programs should not only focus on the specific nutrients, but also on eating behavior. Future researches need to evaluate these patterns in longitudinal studies to understand cause and effect results.

## Supplementary information

**Additional file 1 Table S1**. Description of outcomes and determinants, and measures classification used in this study.

## Data Availability

The datasets used and/or analyzed during the current study are available from the corresponding author on reasonable request.

## Abbreviations

BackPEI: Back Pain and Body Posture Evaluation Instrument
BMI: Body mass index
FIGs: Federal Institutes Games
IPAQ: Physical Activity Questionnaire
PeNSE: Brazilian National School-Based Health Survey
PR: Prevalence ratio PR
