# Supplementary material for: The health-related determinants of eating pattern of high school athletes in Goiás, Brazil
Source: Arch Public Health. 2020 Mar 12;78:9. doi: 10.1186/s13690-020-0396-3 (PMC7066817; doi:10.1186/s13690-020-0396-3)
Supplement: Supplementary file 1 — Additional file 1 Table S1. Description of outcomes and determinants, and measures classification used in this study. [file 13690_2020_396_MOESM1_ESM.docx]

Table S1. Description of outcomes and determinants, and measures classification used in this study.

| **Variables** | **Data collected / Classification** | **Dichotomization** |
| --- | --- | --- |
| *Outcomes* |  |  |
| **Eating pattern** |  |  |
| Have breakfast | 0 / 1-2 / 3-4 / 5-6 / 7 per week | 0-4 days / ≥ 5 days per week (Regular) |
| Vegetable and fruit consumption | 0, 1, 2, 3, 4, 5, 6, 7 per week | 0-4 days / ≥ 5 days per week (Regular) |
| Sweet consumption | 0, 1, 2, 3, 4, 5, 6, 7 per week | 0-4 days / ≥ 5 days per week (Regular) |
| *Health-related determinants* |  |  |
| **Anthropometric** |  |  |
| Body mass index (kg/m^2^) | Normal / Overweight / Obese | Normal weight / Overweight |
| **Body weight control** |  |  |
| Vomiting or using laxatives to lose weight | Yes / No | Yes / No |
| Using pills to lose weight without prescription | Yes / No | Yes / No |
| Using pills to lose improve muscle mass without prescription | Yes / No | Yes / No |
| **Psychosocial** |  |  |
| Body weight self-perception | Severely underweight / Underweight / Normal weight / Overweight / Obese | Normal and not normal |
| Felt lonely in the previous year | Never / Rarely / Sometimes / Most of the time / Always | Never and Rarely / Sometimes or more |
| Lost sleep because of a concern in the previous year | Never / Rarely / Sometimes / Most of the time / Always | Never and Rarely / Sometimes or more |
| Felt intimidated last month | Never / Rarely / Sometimes / Most of the time / Always | Never and Rarely / Sometimes or more |
| **Sedentary Behavior** |  |  |
| Time spent watching TV per day | 0-1 / 2-3 / ≥ 4 hours | 0-1 hours / ≥ 2 hours |
| Time spent using computers per day | 0-1 / 2-3 / ≥ 4 hours | 0-1 hours / ≥ 2 hours |
| Sitting time on a weekday | 0-4 / 5-8 / 9-12 / ≥ 13 hours | 0-8 hours / ≥ 9 hours |
| Sitting time on a weekend day | 0-4 / 5-8 / 9-12 / ≥ 13 hours | 0-8 hours / ≥ 9 hours |
| **Sleeping time** |  |  |
| Time sleeping per night | ≤ 6 / 7 / 8-9 / ≥ 10 hours | ≤ 7 hours / ≥ 8 hours |
| **Meals** |  |  |
| Have breakfast | 0 / 1-2 / 3-4 / 5-6 / 7 per week | 0-4 days / ≥ 5 days per week (Regular) |
| Habit of eating in front of the television | Yes / No | Yes / No |
| **Alcohol and smoking** |  |  |
| Alcohol consumption last month | Yes / No | Yes / No |
| Frequency of alcohol consumption last month | 1-2 days / 3-5 days / 6-9 days / 10-19 days / ≥ 20 days | 1-5 days / ≥ 6 days |
| Doses of alcohol consumption on any occasion last month | < 1 dose / 1 dose / 2 doses / 3 doses / 4 doses / ≥ 5 doses a time | 1-3 doses / ≥ 4 doses a time |
| Smoking habits last month | Yes / No | Yes / No |
| Passive smoking last week | 0 / 1-4 / ≥ 5 days | 0 / ≥ 1 days |
| Smoking parents | Yes / No | Yes / No |
| **Strength** |  |  |
| Trunk strength by weight (Newton) | 1º / 2º / 3º / 4º quartile | Low (1º and 2º) / High (3º and 4º) |
| Handgrip strength by weight (Newton) | 1º / 2º / 3º / 4º quartile | Low (1º and 2º) / High (3º and 4º) |
